# Force/position-based velocity control strategy for the lower limb rehabilitation robot during active training: design and validation

**DOI:** 10.3389/fbioe.2023.1335071

**Published:** 2024-01-08

**Authors:** Junjie Tian, Hongbo Wang, Hao Lu, Yang Yang, Lianqing Li, Jianye Niu, Bo Cheng

**Affiliations:** ^1^ Hebei Provincial Key Laboratory of Parallel Robot and Mechatronic System, Yanshan University, Qinhuangdao, China; ^2^ School of Mechanical Engineering, Yanshan University, Qinhuangdao, China; ^3^ Academy for Engineering and Technology, Fudan University, Shanghai, China; ^4^ Qinhuangdao Hospital of Traditional Chinese Medicine, Qinhuangdao, China

**Keywords:** active training, rehabilitation robot, trajectory tracking, velocity control, active participation

## Abstract

Aiming at the shortcomings of most existing control strategies for lower limb rehabilitation robots that are difficult to guarantee trajectory tracking effect and active participation of the patient, this paper proposes a force/position-based velocity control (FPVC) strategy for the hybrid end-effector lower limb rehabilitation robot (HE-LRR) during active training. The configuration of HE-LRR is described and the inverse Jacobian analysis is carried out. Then, the FPVC strategy design is introduced in detail, including normal velocity planning and tangential velocity planning. The experimental platform for the HE-LRR system is presented. A series of experiments are conducted to validate the FPVC strategy’s performance, including trajectory measurement experiments, force and velocity measurement experiments, and active participation experiments. Experimental studies show that the end effector possesses good following performance with the reference trajectory and the desired velocity, and the active participation of subjects can be adjusted by the control strategy parameters. The experiments have verified the rationality of the FPVC strategy, which can meet the requirements of trajectory tracking effect and active participation, indicating its good application prospects in the patient’s robot-assisted active training.

## 1 Introduction

Stroke is a cerebrovascular disease that seriously endangers human health (Langhorne et al., 2011; Singh et al., 2018). Its high incidence rate and high disability rate have brought heavy burdens to individuals, families and society (Feigin et al., 2009). Epidemiological investigation shows that motor dysfunction is the leading cause of disability after stroke (Zhang et al., 2016; Dulyan et al., 2022). In recent years, many studies have been dedicated to developing rehabilitation robot systems to assist stroke patients in limb rehabilitation training, and a series of research achievements have been made (Krebs et al., 2007; Zhou et al., 2021; Cao et al., 2023).

According to patients’ degree of active participation, training methods are mainly divided into two types: passive training and active training (Shi et al., 2019). The robot guides the patient’s limbs along the required reference trajectory in passive training. It aims to prevent muscle atrophy through repetitive movement (Wu et al., 2022). Passive training is suitable for improving proprioceptive sensitivity around limb joints in the early rehabilitation stage (Chiyohara et al., 2020). In active training, patients are required to complete corresponding tasks within a certain period based on verbal or visual instructions (Sun et al., 2023). Clinical research shows that the patient’s active participation is conducive to motor-related cortical activation and limb rehabilitation (Zheng et al., 2021). The control strategies involved in active training are primarily based on bioelectrical signals and force/torque signals (Zhang et al., 2017).

Two active control strategies utilizing sEMG signals are available for rehabilitation robots: continuous control and triggered control (Meng et al., 2015; Cao et al., 2022). With the continuous control, sEMG signals are used to recognize the limb motion intention, and torque assistance based on this intention is provided for generating the desired motion (Lu et al., 2019). Xie et al. have combined sEMG signals with interaction force to optimize trajectory planning for the rehabilitation robot and planned different periodic trajectories (Xie et al., 2016). Khoshdel et al. developed a neural impedance control strategy to estimate the exerted force using sEMG signals for a single-DOF rehabilitation robot (Khoshdel et al., 2018). Shi et al. proposed a model for predicting the continuous motion of lower limbs for rehabilitation robots (Shi et al., 2020). Their study examined the influence of different muscle types on joint angles as well as the robustness of their prediction model. With the triggered control, the robot begins to provide the assistance when the sEMG signals reach a certain threshold (Artz, 2015). Using the support vector machine classification model, Meng et al. developed a control strategy capable of predicting limb motion intention and triggering robot assistance based on sEMG signals (Meng et al., 2014). Ma et al. used sEMG signals to predict the angles of the hip and knee joints. When the predicted angle values reached the set thresholds, the lower limb rehabilitation robot was triggered to complete the corresponding gait (Ma et al., 2019). An sEMG-based trigger was proposed by Kawamoto et al. for the HAL rehabilitation robot. By providing the patient with motion support, HAL could move the joints in accordance with the movement intention and improve the lower limb’s joint mobility (Kawamoto et al., 2010). Nevertheless, bioelectrical signals used for active control are susceptible to interference and consume considerable time. Implementation and interpretation of this approach are highly dependent on the individual (Taffese, 2017).

Compared with bioelectrical signals, force/torque signals have the advantages of stable performance (Lotti et al., 2022). The active training control strategies based on force/torque information mainly include the impedance control and the hybrid force/position control strategies (Tsoi et al., 2009). The impedance control aims to synchronously adjust motion and force by establishing an appropriate interaction relationship (Zhou et al., 2021). Huo et al. developed an impedance modulation method for the exoskeleton robot, which can provide balance assistance during the switch between sitting and standing (Huo et al., 2022). Mokhtari et al. proposed a hybrid optimal sliding mode impedance control method and compared the performance with that of the traditional sliding mode controller in the lower limb exoskeleton system (Mokhtari et al., 2021). Tran et al. designed a fuzzy rule-based impedance control strategy that can adjust the impedance coefficients between the robot and the lower limb under various walking speeds (Tran et al., 2016). The hybrid position/force controller is intended for both position and force trajectory tracking (Navvabi and Markazi, 2019). Bernhardt et al. proposed a hybrid control strategy for the rehabilitation robot Lokomat. In the swing phase, the rehabilitation robot was controlled by force so the patient could walk independently. In the stance phase, the control software switched to position control to guide the limb to move (Bernhardt et al., 2005). Ju et al. developed a hybrid position/force controller for the rehabilitation robot combined with fuzzy logic to track the desired force along the preset motion direction (Ju et al., 2005). Valera et al. developed a hybrid control scheme based on the position/force information, which makes it possible to perform different lower limb rehabilitation exercises (Valera et al., 2017). However, due to the position/force dynamic relationship being adjusted to increase robot compliance in impedance control, it increases the difficulty of guaranteeing the trajectory tracking effect of the robot in lower limb rehabilitation training (Lv et al., 2017). The common position/force hybrid control strategy allows patients to bear a certain amount of resistance close to the preset trajectory, which limits the active participation of patients (Rivas-Blanco et al., 2013).

Aiming at the shortcomings of most existing control strategies for lower limb rehabilitation robots that are difficult to guarantee trajectory tracking effect and active participation of the patient, a force/position-based velocity control (FPVC) strategy is proposed for the hybrid end-effector lower limb rehabilitation robot (HE-LRR) in this paper. On one hand, HE-LRR has the advantages of a large workspace and strong bearing capacity and is also suitable for experimental verification on subjects with different body dimensions. On the other hand, HE-LRR can guide the lower limbs to perform three-dimensional spatial movements, achieving various typical lower limb rehabilitation exercises such as MOTOmed therapy and continuous passive motion (CPM) therapy. Experimental studies have been conducted to verify the rationality of the FPVC strategy under MOTOmed and CPM modes. This paper is organized as follows. In the Materials and Methods section, the configuration of HE-LRR is introduced and the FPVC strategy design is proposed. Then the experimental platform is described. In the Results section, the validation experiments are conducted, including trajectory measurement experiments, force and velocity measurement experiments, and active participation experiments. In the Conclusions and Discussion section, the summary and prospect of the FPVC strategy are presented.

## 2 Materials and methods

### 2.1 Configuration of HE-LRR

**FIGURE 1:**
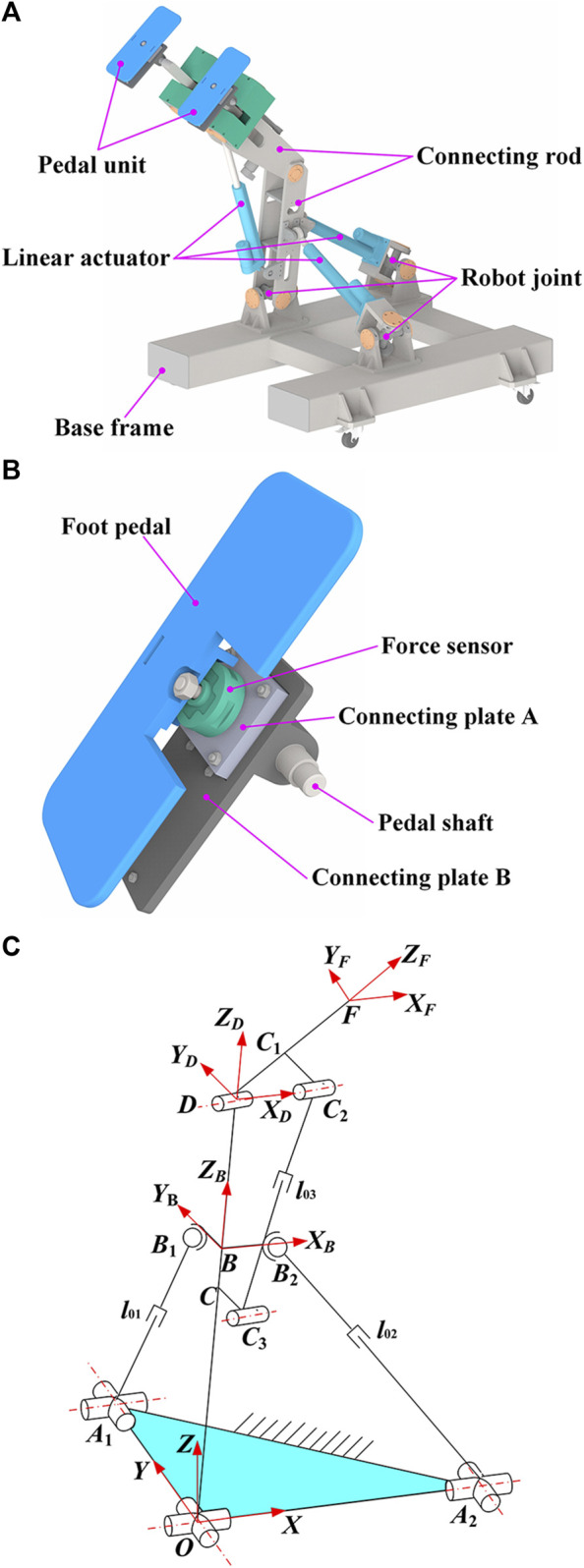
Configuration of HE-LRR. **(A)** Overall structure of the robot. **(B)** Detailed structure of the pedal unit. **(C)** Schematic diagram of robot configuration.

HE-LRR consists of a base frame, connecting rods, linear actuators, robot joints, and pedal units, as shown in Figure 1A. The pedal unit is the end effector of HE-LRR, consisting of a foot pedal, a force sensor, connecting plates, and a pedal shaft, as shown in Figure 1B.

Figure 1C shows the robot configuration diagram. The origin of the fixed coordinate system {*O*-*XYZ*} lies at the intersection of the two rotational auxiliary axes of the universal joint. The *X*-axis coincides with one axis of the universal joint and along the *OA*_2_ direction. The *Y*-axis coincides with another axis of the universal joint and along the *OA*_1_ direction. The direction of the *Z*-axis is determined by the right-hand screw rule. Moving coordinate system {*B*-*X*_*B*_ *Y*_*B*_ *Z*_*B*_} has its origin at the *OD* rod, *X*_*B*_ axis along the *BB*_2_ direction, and *Y*_*B*_ axis along the *BB*_1_ direction. The moving coordinate system {*D*-*X*_*D*_ *Y*_*D*_ *Z*_*D*_} is established with the *X*_*D*_ axis along the axis of the revolute joint *D* and the *Z*_*D*_ axis along the *OD* direction. Point *F* represents the midpoint of the robot end effectors (pedal units). The *X*_*F*_ axis of the moving coordinate system {*F*-*X*_*F*_ *Y*_*F*_ *Z*_*F*_} is parallel to the *X*_*D*_ direction, and the *Z*_*F*_ axis is along the *DF* direction.

### 2.2 Inverse Jacobian analysis

In this subsection, the parameter symbols and descriptions of the robot configuration are shown in Table 1. According to the geometric relationships in Figure 1C, the coordinates of point *F* can be expressed as follows:

$$\left\{\begin{array}{l} F_{x}=l_{2}\sin\beta \cos\left(\alpha +\gamma \right)+l_{1}\cos\alpha \sin\beta \\ F_{y}=-l_{2}\sin\left(\alpha +\gamma \right)-l_{1}\sin\alpha \\ F_{z}=l_{2}\cos\beta \cos\left(\alpha +\gamma \right)+l_{1}\cos\alpha \cos\beta \end{array}\right.$$ (1)

**TABLE 1:** Parameter symbols and descriptions of the robot configuration.

Symbol	Description
*l*_1_	Length of *OD*
*l*_2_	Length of *DF*
*l*_01_	Length of *A*_1_ *B*_1_
*l*_02_	Length of *A*_2_ *B*_2_
*l*_03_	Length of *C*_2_ *C*_3_
*a*_1_	Length of *OA*_1_
*a*_2_	Length of *OA*_2_
*b*_1_	Length of *BB*_1_
*b*_2_	Length of *BB*_2_
*a*_3_	Length of *CC*_3_
*b*_3_	Length of *C*_1_ *C*_2_
*l*_*OB*_	Length of *OB*
*m*_1_	Length of *CD*
*m*_2_	Length of *C*_1_ *D*
*α*	The angle of {*D*-*X*_D_ *Y*_D_ *Z*_D_} relative to {*O*-*XYZ*} around the *X*-axis
*β*	The angle of {*D*-*X*_D_ *Y*_D_ *Z*_D_} relative to {*O*-*XYZ*} around the *Y*-axis
*γ*	The angle of {*F*-*X*_F_ *Y*_F_ *Z*_F_} relative to {*D*-*X*_D_ *Y*_D_ *Z*_D_} around the *X*_*D*_ axis

According to Eq. 1, the rotation angles *α*, *β* and *γ* can be expressed as:

$$\left\{\begin{array}{l} \alpha =-\arccos\left(\frac{{l_{1}}^{2}+F_{x}^{2}+F_{y}^{2}+F_{z}^{2}-{l_{2}}^{2}}{2l_{1}\sqrt{F_{x}^{2}+F_{y}^{2}+F_{z}^{2}}}\right)-\arctan\left(\frac{F_{y}}{F_{z}}\cos\beta \right)\\ \beta =\arctan\left(\frac{F_{x}}{F_{z}}\right)\\ \gamma =\arccos\frac{{F_{x}}^{2}+{F_{y}}^{2}+{F_{z}}^{2}-{l_{2}}^{2}-{l_{1}}^{2}}{2l_{1}l_{2}} \end{array}\right.$$ (2)

Taking the derivative of time on both sides of Eq. 2, the mapping relationship between the angular velocities and the velocity components of the robot end effector can be expressed in the following matrix form:

$$\left[\begin{array}{c} \dot{\alpha }\\ \dot{\beta }\\ \dot{\gamma } \end{array}\right]=J_{1}\left[\begin{array}{c} {\dot{F}}_{x}\\ {\dot{F}}_{y}\\ {\dot{F}}_{z} \end{array}\right]=\left[\begin{array}{ccc} \frac{\partial \alpha }{\partial F_{x}} & \frac{\partial \alpha }{\partial F_{y}} & \frac{\partial \alpha }{\partial F_{z}}\\ \frac{\partial \beta }{\partial F_{x}} & \frac{\partial \beta }{\partial F_{y}} & \frac{\partial \beta }{\partial F_{z}}\\ \frac{\partial \gamma }{\partial F_{x}} & \frac{\partial \gamma }{\partial F_{y}} & \frac{\partial \gamma }{\partial F_{z}} \end{array}\right]\left[\begin{array}{c} {\dot{F}}_{x}\\ {\dot{F}}_{y}\\ {\dot{F}}_{z} \end{array}\right]$$ (3)

where ***J***_1_ is the velocity Jacobian matrix between the angular velocities and velocity components of the robot end effector.

Establish the following closed-loop vector equation in the fixed coordinate system {*O-XYZ*}

lOB+RBOni=mi+AiBii=1,2 (4)

where RBO is the rotation matrix from {*B-X*_*B*_ *Y*_*B*_ *Z*_*B*_} to {*O*-*XYZ*}, $n_{i}$ is the position vector of *B*_*i*_ in the coordinate system {*B-X*_*B*_ *Y*_*B*_ *Z*_*B*_}, $m_{i}$ is the position vector of *A*_*i*_ in the coordinate system {*O-XYZ*}, $l_{OB}$ and $A_{i}B_{i}$ are the vectors of *OB* and *A*_*i*_ *B*_*i*_ in the fixed coordinate system.

Substituting mechanical parameters into Eq. 4 and simplifying to get expressions of the linear actuator lengths *l*_01_ and *l*_02_

$$\left\{\begin{array}{l} l_{01}=\sqrt{a_{1}^{2}+b_{1}^{2}+l_{OB}^{2}-1a_{1}b_{1}\cos\alpha +2l_{OB}a_{1}\sin\alpha }\\ l_{02}=\sqrt{a_{2}^{2}+b_{2}^{2}+l_{OB}^{2}-1a_{2}b_{2}\cos\beta +2l_{OB}a_{1}\sin\beta } \end{array}\right.$$ (5)

According to the Cosine Theorem, we can get Eq. 6

$$\cos∠C_{2}DC_{3}=\frac{b_{3}^{2}+m_{2}^{2}+m_{1}^{2}+a_{3}^{2}-l_{03}^{2}}{2\sqrt{b_{3}^{2}+m_{2}^{2}}\sqrt{m_{1}^{2}+a_{3}^{2}}}$$ (6)

*γ* angle can be solved as Eq. 7

$$\gamma =\pi -\arccos\frac{b_{3}^{2}+m_{2}^{2}+a_{3}^{2}+m_{1}^{2}-l_{03}^{2}}{2\sqrt{b_{3}^{2}+m_{2}^{2}}\sqrt{a_{3}^{2}+m_{1}^{2}}}$$ (7)

Hence, the linear actuator length *l*_03_ can be expressed as

$$l_{03}=\sqrt{a_{3}^{2}+b_{3}^{2}+m_{1}^{2}+m_{2}^{2}+2\sqrt{m_{1}^{2}+a_{3}^{2}}\sqrt{m_{2}^{2}+b_{3}^{2}}\cos\left(\begin{array}{c} \gamma +\arctan\frac{b_{3}}{m_{2}}\\ +\arctan\frac{a_{3}}{m_{1}} \end{array}\right)}$$ (8)

The relationship between linear actuator velocities and angular velocities can be obtained by taking the derivative of time on both sides of Eqs 5, 8:

$$\left\{\begin{array}{l} {\dot{l}}_{01}=\frac{a_{1}l_{OB}\cos\alpha +a_{1}b_{1}\sin\alpha }{l_{01}}\dot{\alpha }\\ {\dot{l}}_{02}=\frac{a_{2}l_{OB}\sin\alpha \sin\beta }{l_{02}}\dot{\alpha }\\ +\frac{a_{2}b_{2}\sin\beta -a_{2}l_{OB}\cos\alpha \cos\beta }{l_{02}}\dot{\beta }\\ {\dot{l}}_{03}=-\frac{\sin\left(\begin{array}{l} \gamma +\arctan\frac{b_{3}}{m_{2}}\\ +\arctan\frac{a_{3}}{m_{1}} \end{array}\right)\sqrt{{a_{3}}^{2}+{m_{1}}^{2}}\sqrt{{b_{3}}^{2}+{m_{2}}^{2}}}{l_{03}}\dot{\gamma } \end{array}\right.$$ (9)

***J***_2_ is used to represent the Jacobian matrix between the linear actuator velocities and angular velocities. Eq. 9 can be written in the following matrix form:

$$\left[\begin{array}{c} {\dot{l}}_{01}\\ {\dot{l}}_{02}\\ {\dot{l}}_{03} \end{array}\right]=J_{2}\left[\begin{array}{c} \dot{\alpha }\\ \dot{\beta }\\ \dot{\gamma } \end{array}\right]=\left[\begin{array}{ccc} J_{11} & 0 & 0\\ J_{21} & J_{22} & 0\\ 0 & 0 & J_{33} \end{array}\right]\left[\begin{array}{c} \dot{\alpha }\\ \dot{\beta }\\ \dot{\gamma } \end{array}\right]$$ (10)

where

$$\left\{\begin{array}{l} J_{11}=\frac{a_{1}l_{OB}\cos\alpha +a_{1}b_{1}\sin\alpha }{l_{01}}\\ J_{21}=\frac{a_{2}l_{OB}\sin\alpha \sin\beta }{l_{02}}\\ J_{22}=\frac{a_{2}b_{2}\sin\beta -a_{2}l_{OB}\cos\alpha \cos\beta }{l_{02}}\\ J_{33}=-\frac{\sin\left(\begin{array}{l} \gamma +\arctan\frac{b_{3}}{m_{2}}\\ +\arctan\frac{a_{3}}{m_{1}} \end{array}\right)\sqrt{{a_{3}}^{2}+{m_{1}}^{2}}\sqrt{{b_{3}}^{2}+{m_{2}}^{2}}}{l_{03}} \end{array}\right.$$

The inverse Jacobian matrix ***J***_*i*_ can be used to represent the mapping relationship between the linear actuator velocities and the velocity components of the robot end effector. Combined with Eqs 3, 10, we can obtain Eq. 11:

$$\left[\begin{array}{c} {\dot{l}}_{01}\\ {\dot{l}}_{02}\\ {\dot{l}}_{03} \end{array}\right]=J_{i}\left[\begin{array}{c} {\dot{F}}_{x}\\ {\dot{F}}_{y}\\ {\dot{F}}_{z} \end{array}\right],J_{i}=J_{1}J_{2}$$ (11)

### 2.3 FPVC strategy design

**FIGURE 2:**
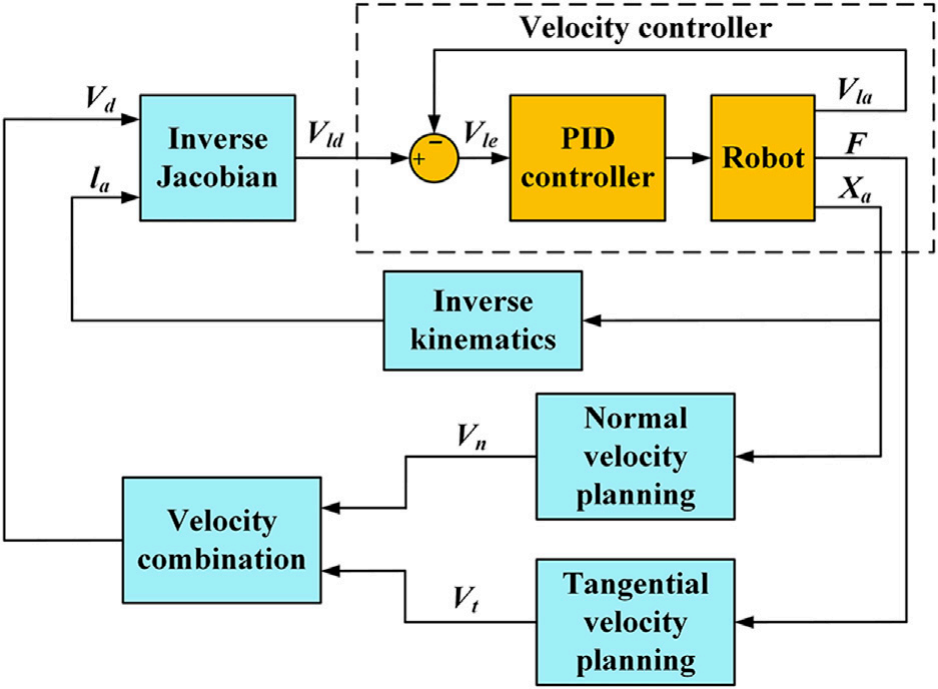
FPVC strategy diagram for HE-LRR.

Figure 2 shows the FPVC strategy diagram for HE-LRR. The end effector’s actual three-dimensional position coordinate information *X*_*a*_ is used to plan the normal velocity (NV) *V*_*n*_ of HE-LRR, and man-machine contact force (MCF) *F* is used to plan the tangential velocity (TV) *V*_*t*_ of HE-LRR. The NV and TV are combined as the end effector’s desired velocity *V*_*d*_. The actual position of each linear actuator *l*_a_ is calculated by inverse kinematics. Combined with the desired velocity *V*_*d*_ and the actual linear actuator position *l*_a_, the desired velocity of each linear actuator *V*_*ld*_ is calculated by inverse Jacobian and is sent to the velocity controller of the FPVC strategy. The calculation process of inverse kinematics is shown in the literature (Wang et al., 2022). NV planning and TV planning are introduced in this subsection in detail.

HE-LRR assists the patient’s lower limbs in performing rehabilitation exercises under the constraint trajectory through end traction, so it is essential that the end effector can move along the reference trajectory in space. When the end effector deviates from the reference trajectory, the desired NV is planned to reduce the deviation. *P*_1_ is the actual end point of the end effector of HE-LRR, and *P*_2_ is the closest point on the reference trajectory to point *P*_1_. The desired NV direction is along the direction of *P*_1_ *P*_2_ and points towards *P*_2_.

The end effector’s desired NV can be calculated by Eq. 12:

$$V_{n}=k_{n}d$$ (12)

where *k*_*n*_ is the NV coefficient, and *d* is the shortest distance from point *P*_1_ to the reference trajectory.

The mapping function between TV and MCF is planned as a piecewise function, including the initial segment sub-function, linear segment sub-function and parabolic segment sub-function. When MCF is less than the initial threshold *F*_*i*_, it is considered that MCF is caused by random factors such as mechanical jitter, and cannot represent the patient’s active intention, and the desired TV is equal to zero. When the MCF exceeds the initial threshold *F*_*i*_ and falls below the linear threshold *F*_*l*_, it is considered that MCF can reflect the patient’s movement intention. The mapping function is planned as a linear correlation between the desired TV and MCF. When MCF exceeds the linear threshold *F*_*l*_, the slope of the mapping function needs to decrease based on safety consideration, and the mapping relationship between TV and MCF is planned as a parabolic sub-function.

According to the above parameters and settings, the initial segment sub-function is:

$$V_{t}=0\left(F\leq F_{i}\right)$$ (13)

The linear segment sub-function is:

$$V_{t}=k_{l}F-k_{l}F_{i}\left(F_{i}<F\leq F_{l}\right)$$ (14)

where *k*_*l*_ represents the linear segment slope.

The linear threshold *F*_*l*_ can be expressed as follows:

$$F_{l}=\frac{V_{lm}+k_{l}F_{i}}{k_{l}}$$ (15)

where *V*_*lm*_ represents the maximum linear velocity.

The parabolic equation whose focus is on the *F*-axis is chosen for the planning of the third segment sub-function. The parabolic sub-function can be written as:

$$V_{t}=\sqrt{2p\left(F-q\right)}$$ (16)

where *q* is the *F*-axis translation distance, and *p* represents the distance from the focus to the directrix of the parabola.

To meet the piecewise function’s continuity requirement, the point (*F*_*l*_, *V*_*lm*_) is the intersection point of the linear segment and the parabolic segment, thus:

$$V_{lm}=\sqrt{2p\left(F_{l}-q\right)}$$ (17)

The parabolic slope at the point (*F*_*l*_, *V*_*lm*_) is set to half the linear slope, thus:

$$\frac{k_{l}}{2}=\frac{p}{\sqrt{2p\left(F_{l}-q\right)}}$$ (18)

Combined with Eqs 15–18, the parabolic segment sub-function can be expressed as:

$$V_{t}=\sqrt{k_{l}V_{lm}\left(F-F_{i}\right)}\;\left(F>F_{l}\right)$$ (19)

Combined with Eqs 13, 14, 19, the piecewise function can be expressed as:

$$V_{t}=\left\{\begin{array}{cc} 0 & F\leq F_{i}\\ k_{l}F-k_{l}F_{i} & F_{i}<F\leq \frac{V_{lm}+k_{l}F_{i}}{k_{l}}\\ \sqrt{k_{l}V_{lm}\left(F-F_{i}\right)} & F>\frac{V_{lm}+k_{l}F_{i}}{k_{l}} \end{array}\right.$$ (20)

It can be seen from Eq. 20 that the mapping function between TV and MCF can be determined by three parameters, including the initial threshold *F*_*i*_, the linear segment slope *k*_*l*_, and the maximum linear velocity *V*_*lm*_.

### 2.4 Experimental platform

**FIGURE 3:**
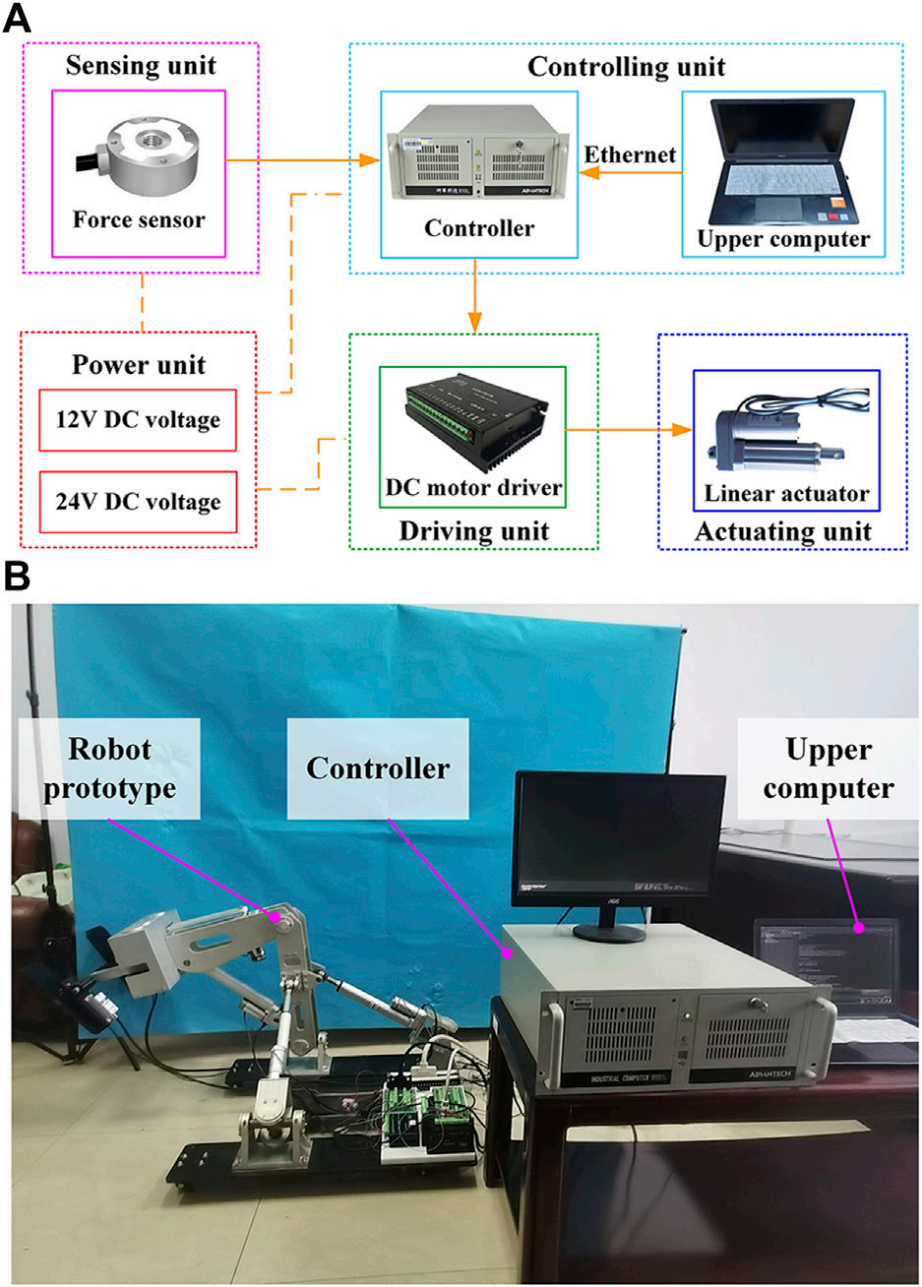
Experimental platform. **(A)** Block diagram of the robot control system. **(B)** Physical picture of the robot prototype and the control system.

Figure 3A illustrates the block diagram of the lower limb rehabilitation robot’s control system, which includes the controlling, sensing, driving, actuating, and power units. A personal computer (Advantech, IPC610, CN) serves as the controller. In addition to receiving commands from the upper computer (Dell, Vostro 5370, USA), the IPC can also receive signals from force sensors (HUILIZHI, LZ-SWF40, 0–300 N, ±0.3%F.S., CN) and encoders. The motor drivers (Magicon Intelligent, MC-FBLD-6600, 9–36 V, 12 A, CN) receive commands from the controller to accomplish the telescopic movement of the linear actuators (YCMC, LEC606, 210 mm, 0–450 N, CN). An incremental encoder records the DC motor’s actual position as it moves to facilitate the linear actuator’s velocity closed-loop control. The angle sensors, encoders, and motor drivers are powered by the power unit that supplies 12 V or 24 V DC voltage.

As shown in Figure 3B, the prototype of HE-LRR has been manufactured, and the control system has been built. The robot’s base frame is equipped with casters with brakes to facilitate robot movement and improve stability. Rehabilitation training is performed with the patient’s feet on the pedal units. According to the procedure (CRRC-IEC-RF-SC-005-01) approved by the China Rehabilitation Research Center, three healthy subjects were recruited to participate in the experiments. Basic information about the subjects is presented in Table 2. During the experiments, none of the subjects reported discomfort.

**TABLE 2:** Basic information on healthy subjects.

Number	Height (mm)	Weight (kg)	Thigh length (mm)	Calf length (mm)
1	1720	75	430	400
2	1670	78	405	385
3	1690	72	415	400

## 3 Results

To validate the feasibility of the FPVC strategy, trajectory measurement experiments, force and velocity measurement experiments, and active participation experiments are carried out in this section.

### 3.1 Trajectory measurement experiments

**FIGURE 4:**
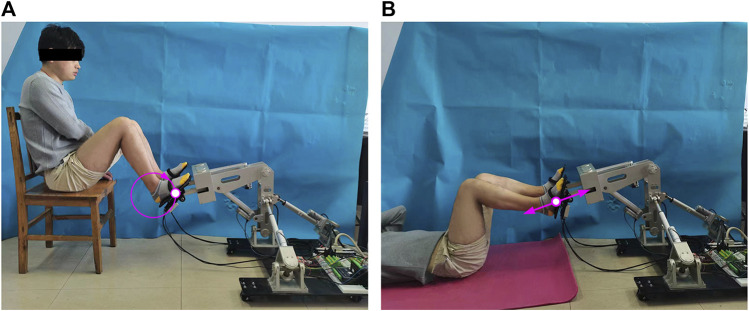
Field diagram of trajectory measurement experiments of HE-LRR. **(A)** MOTOmed mode. **(B)** CPM mode.

The active training based on the FPVC strategy is carried out under the constraint trajectory, which makes it possible for patients to obtain a large range of joint activities. The trajectory measurement experiments of HE-LRR are carried out under MOTOmed mode (Figure 4A) and CPM mode (Figure 4B). The constraint trajectories for the above two modes are a circular trajectory and a linear trajectory, respectively. The subject’s feet are connected with the end effector through Velcro tapes. During the experiment, the actual positions of the linear actuators are recorded, and the actual end position of HE-LRR can be calculated through the forward kinematics of the robot. Each group of experiments was conducted for 10 min. In subsections 3.1 and 3.2, 10 s of data were displayed to more clearly represent the results.

**FIGURE 5:**
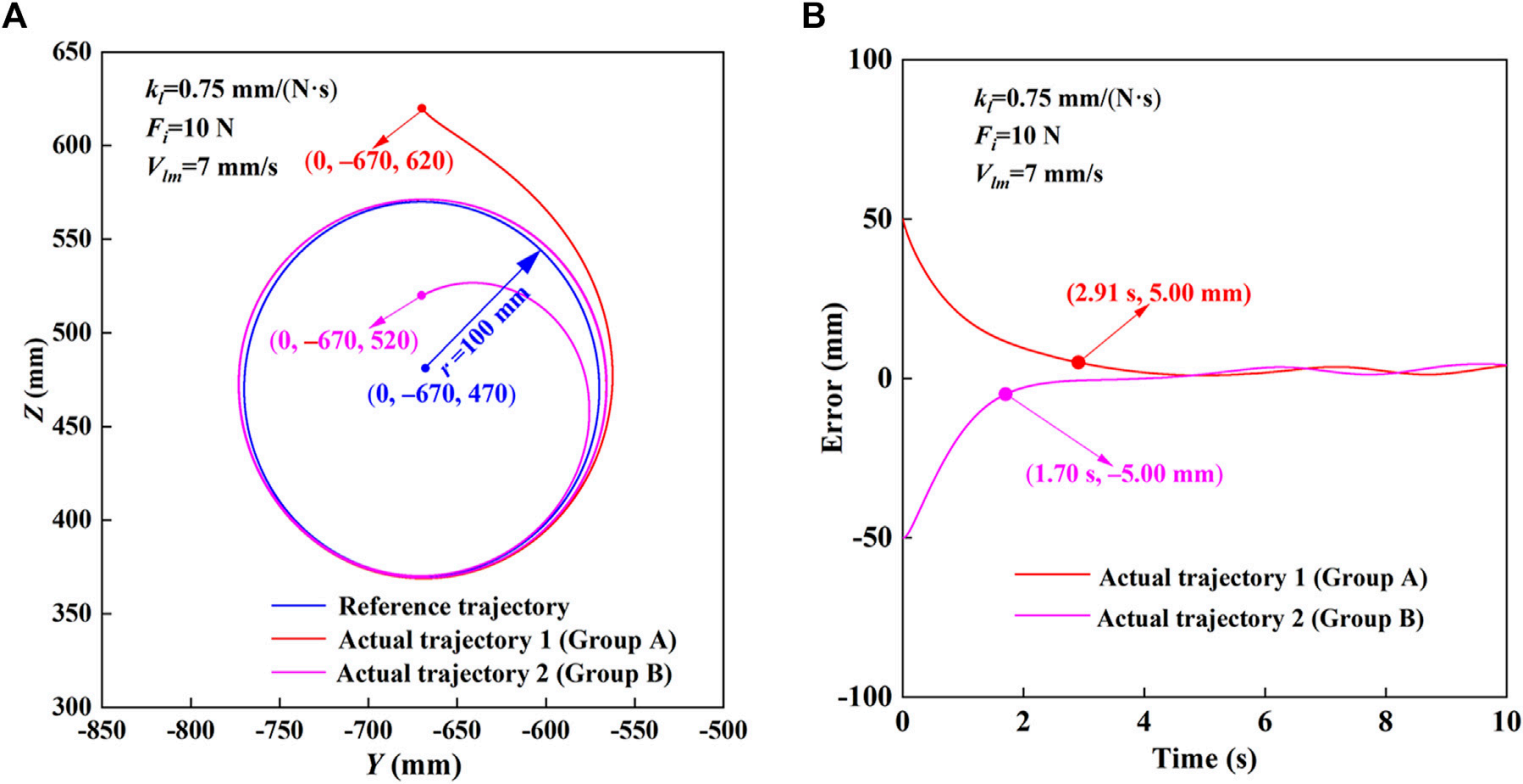
Trajectory measurement experimental results of HE-LRR in MOTOmed mode. **(A)** Comparison of the reference trajectory and actual trajectory. **(B)** Actual position errors.

The trajectory measurement experimental results of HE-LRR in MOTOmed mode are shown in Figure 5. The reference trajectory parameters are set as shown in Figure 5A: the center coordinates (*x*_0_, *y*_0_, *z*_0_)=(0, −670, 470), the radius is 100.00 mm. When the initial position is outside the circular trajectory, the initial point is set to (*x*_0_, *y*_0_, *z*_0_)=(0, −670, 620). When the initial position is inside the circular trajectory, the initial point is set to (*x*_0_, *y*_0_, *z*_0_)=(0, −670, 520). It can be seen that during the experiment, the end position of the robot quickly approaches the reference trajectory first, and then the approaching velocity slows down. Finally, the end effector’s actual trajectory has a good coincidence degree with the reference trajectory. The minimum distance between the end effector’s actual position and the reference trajectory is defined as the actual position error of the robot. Figure 5B shows the robot’s actual position errors under MOTOmed mode. The position errors of the robot are different due to the difference in the starting point. The initial position errors of experimental Group A and Group B are 50 mm and −50 mm, respectively. The positive error value indicates that the initial point is outside the circular trajectory, and the negative value indicates that the initial point is inside the circular trajectory. At about 2.91 s, the position error of experimental Group A decreases to 5.00 mm. At about 1.70 s, the position error of experimental Group B becomes −5.00 mm. After about 4 s, the end error of the robot decreases to a small range, which suggests that the trajectory tracking effect of the end effector shows good accuracy and stability under different initial position errors.

**FIGURE 6:**
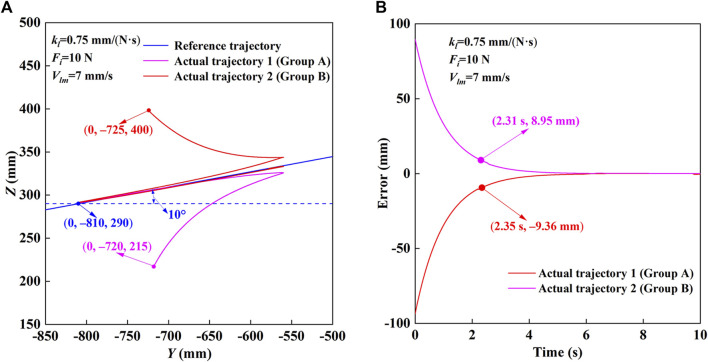
Trajectory measurement experimental results of HE-LRR in CPM mode. **(A)** Comparison of the reference trajectory and actual trajectory. **(B)** Actual position errors.

The trajectory measurement experimental results of HE-LRR in CPM mode are shown in Figure 6. The reference trajectory parameters are set as shown in Figure 6A: the linear trajectory passes through (*x*_0_, *y*_0_, *z*_0_)=(0, −810, 290), and the inclination angle is 10°. When the starting point is above the linear trajectory, the initial point is set as (*x*_0_, *y*_0_, *z*_0_)=(0, −725, 400). When the starting point is below the linear trajectory, the initial point is set as (*x*_0_, *y*_0_, *z*_0_)=(0, −720, 215). It can be seen that the end position of the robot approaches the reference trajectory quickly initially. After the switch from forward motion to backward motion, the end position of the robot still gets close to the reference trajectory. Finally, the actual trajectory has a good coincidence with the reference trajectory. According to Figure 6B, when the starting point is above the linear trajectory, the initial position error is −93.57 mm, and after 2.35 s, the position error becomes −9.36 mm (approximately 10% of the initial position error), and the final position error is in a small range. When the starting point is below the linear trajectory, the initial position error is 89.48 mm. After 2.31 s, the position error decreases to 8.95 mm (approximately 10% of the initial position error), and the final position error is in a small range. In conclusion, the robot based on the FPVC strategy can realize the rehabilitation training of the predetermined trajectory with good accuracy and stability under the MOTOmed and CPM modes.

### 3.2 Force and velocity measurement experiments

In active training, the robot system recognizes the motion intention of the patient by detecting the force applied at the end effector, and assists the lower limb in realizing the rehabilitation training through the actuating unit. The parameter settings of the circular trajectory and the linear trajectory are consistent with those in Section 5.1. The control strategy parameters are set as follows: the initial threshold *F*_*i*_ = 10 N, the maximum linear velocity *V*_*lm*_ = 7 mm/s, and the linear segment slope *k*_*l*_ = 0.75 mm/(N·s). Based on the forward kinematics of the robot, the end effector’s actual position can be calculated from the actual positions of linear actuators. After the differential calculation, the end effector’s actual velocity can be obtained.

**FIGURE 7:**
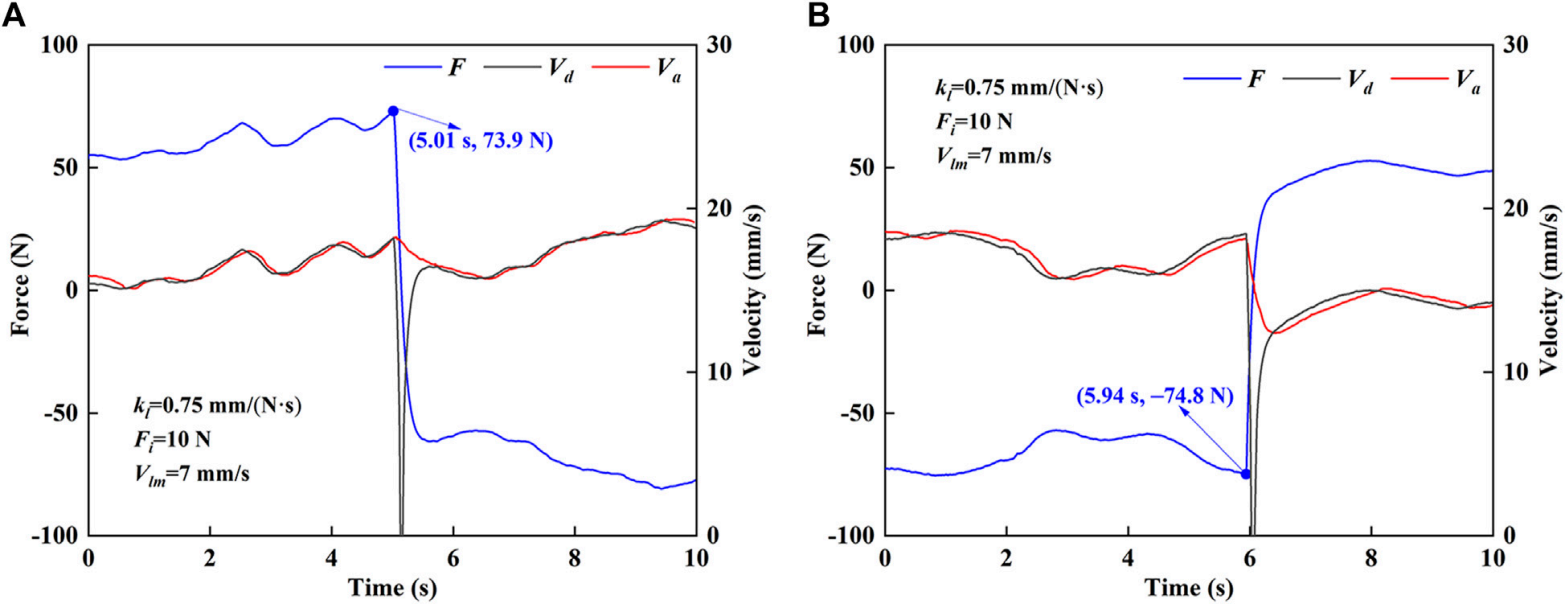
Force and velocity results of HE-LRR in MOTOmed mode. **(A)** Clockwise motion. **(B)** Counterclockwise motion.

Figure 7 shows the experimental results of the force and velocity of the robot in MOTOmed mode. In MOTOmed mode, clockwise and counterclockwise motions are studied, respectively. In the clockwise motion (Figure 7A), when the MCF value is positive, the desired velocity is also non-negative and the fluctuation trend of the desired velocity is consistent with MCF. When the MCF value is negative, the desired velocity is non-negative and the fluctuation trend of the desired velocity is opposite to that of MCF. After 5.01 s, the MCF rapidly changes from compression force (73.9 N) to tension force. At this time, the desired velocity rapidly decreases to 0 mm/s and then rapidly increases. In the counterclockwise motion (Figure 7B), when the MCF value is negative, the desired velocity is non-negative and the fluctuation trend of the desired velocity is opposite to that of the MCF. When the MCF value is positive, the desired velocity is non-negative and the desired velocity and MCF have the same fluctuation trend. From 5.94 s, the MCF quickly changes from tension force (−74.8 N) to compression force, and the desired velocity shows a changing law of rapid decrease and rapid increase. When the desired velocity change rate is low, the end effector’s actual velocity can better follow the desired velocity. When the desired velocity curve has a significant mutation, the changing trend of the actual velocity is quite different and the velocity change is relatively slow. Since it is expected to avoid the velocity mutation during the rehabilitation training, it is beneficial that the actual velocity of the robot can keep relatively stable.

**FIGURE 8:**
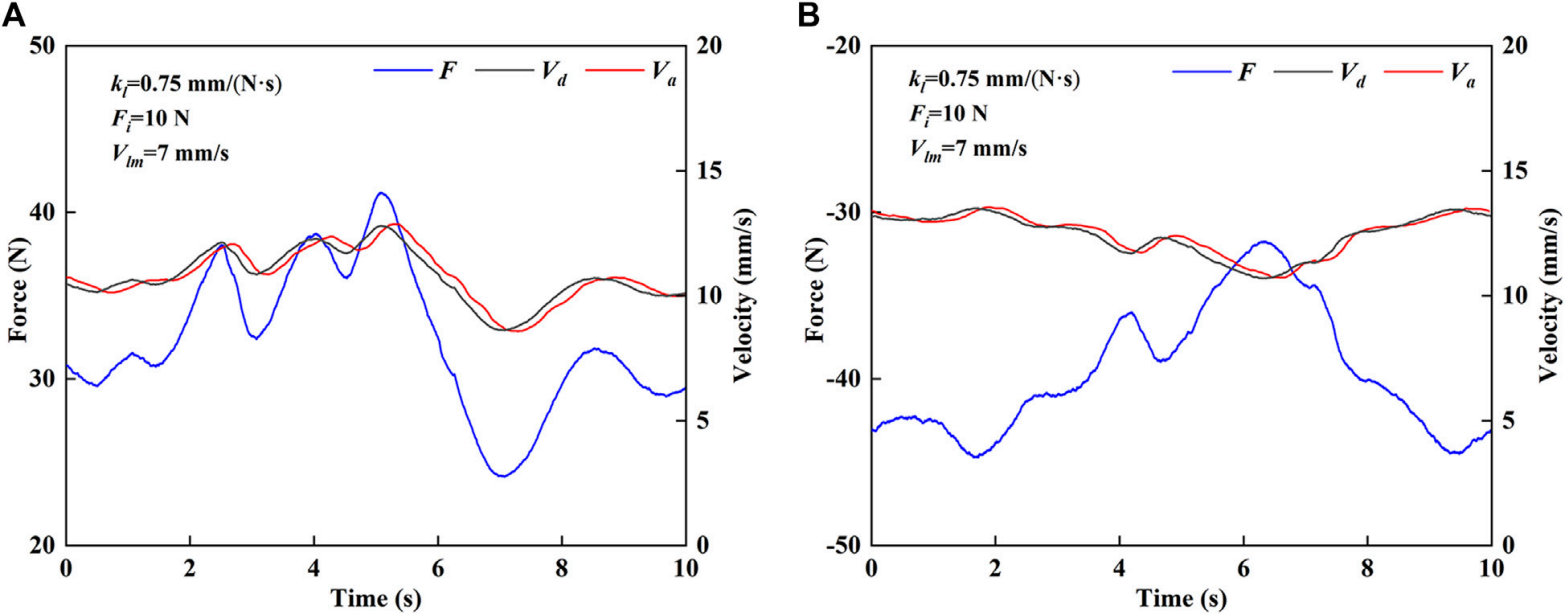
Force and velocity results of HE-LRR in CPM mode. **(A)** Forward motion. **(B)** Backward motion.

Figure 8 shows the experimental results of the force and velocity of the robot in CPM mode. In CPM mode, it is divided into forward motion and backward motion for research. As shown in Figure 8A, in the forward motion, the value of MCF is positive, and the desired velocity is non-negative. The desired velocity and the MCF show similar fluctuation trends. As shown in Figure 8B, in the backward motion, the MCF value is negative, and the desired velocity is non-negative. The desired velocity and the MCF show opposite fluctuation trends. It can be seen that the end effector’s actual velocity has good following ability to the desired velocity in both forward and backward motions, which indicates that the robot motion is very sensitive to the change of the MCF, and can adapt to the active movement intention of the subject.

### 3.3 Active participation experiments

In order to study the FPVC strategy parameters’ effects on the active participation of subjects, this section conducts experimental research on active participation. We recruited three healthy subjects to participate in the MOTOmed and CPM modes. All subjects agreed to include personal data in the study before the experiments. Each subject participated in 30 active training tasks under different control strategy parameters.

During active participation experiments, subjects were required to complete a certain number of tasks in 10 minutes. For the MOTOmed mode, eight cycles of training needed to be completed per minute; For the CPM mode, it was required to complete ten cycles of training per minute, and each subject could have a rest and physical recovery after completing each task. After completing the training task, the subject was asked to take a questionnaire on the subjective feeling and the participation score. When the subject’s subjective feeling was boredom, relaxation, excitement, stress, or frustration, the corresponding participation score was 1, 2, 3, 4, or 5, respectively.

During the experiment, the MCF signal collected by the force sensor was filtered by the Kalman filter, and the objective feature values of root mean square (RMS), mean absolute value (MAV), variance (VAR), and zero crossing (ZC) were extracted from the processed MCF signal. RMS and MAV are statistics which can reflect the MCF signal’s effective value and average strength. VAR can provide information regarding the signal’s power. ZC represents the number of times the signal crosses the zero line, reflecting the number of times the MCF signal switches between tension force and compression force and can be used to calculate the number of training cycles. The additional threshold judgment is introduced to reduce the impact of signal noise on the ZC. The feature values can be calculated according to Eq. 21:

$$\left\{\begin{array}{l} \text{MAV}=\frac{1}{N}\sum_{i=1}^{N}\left|x_{i}\right|\\ \text{RMS}=\sqrt{\frac{\sum_{i=1}^{N}x_{i}^{2}}{N}}\\ \text{VAR}=\frac{1}{N-1}\sum_{i=1}^{N}x_{i}^{2}\\ \text{ZC}=\sum_{i=1}^{N-1}f\left(i\right)\\ f\left(i\right)=\left\{\begin{array}{cc} 1 & x_{i}x_{i+1}<0,\text{and }\left|x_{i}-x_{i+1}\right|>\text{threshold}\\ 0 & \text{otherwise} \end{array}\right. \end{array}\right.$$ (21)

where *x*_*i*_ represents the *i*th value of the MCF signal, and *N* represents the number of the data values.

**FIGURE 9:**
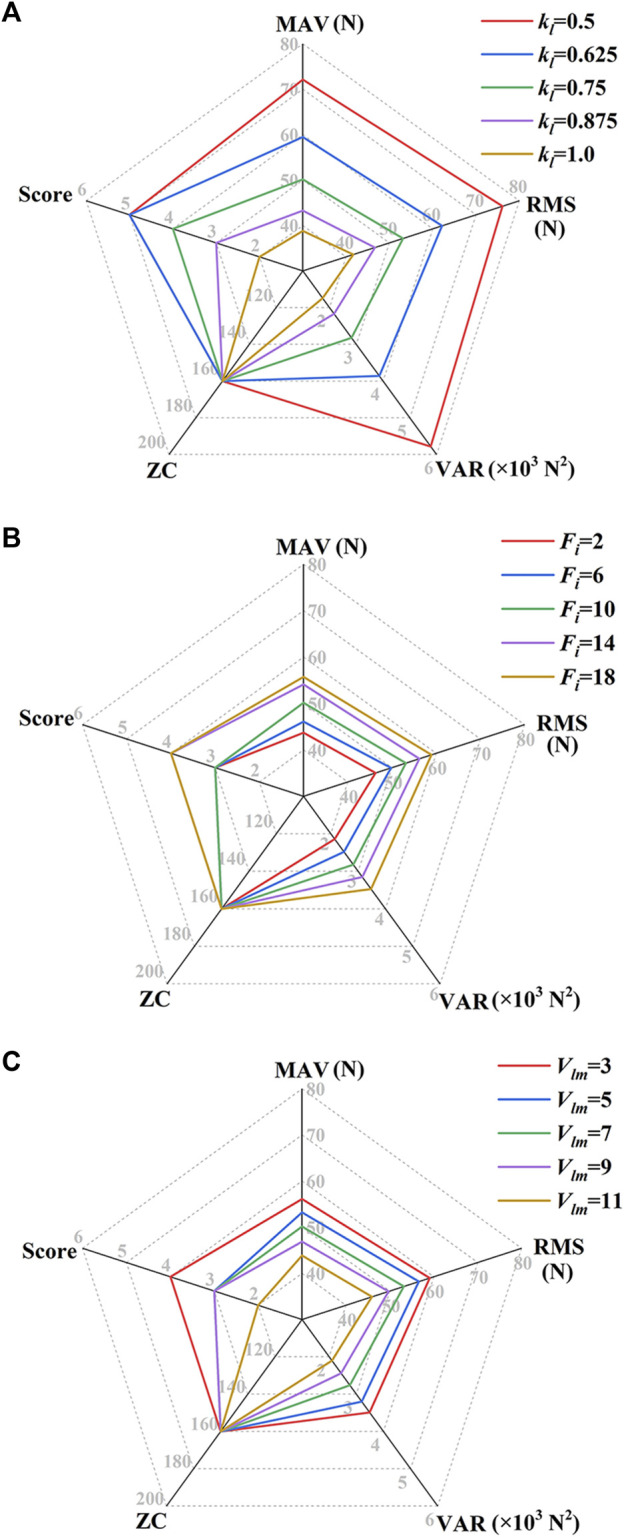
MCF’s feature values and the participation score of Subject No. 1 in the MOTOmed mode under different FPVC strategy parameters. **(A)** *F*_*i*_ = 10 N, *V*_*lm*_ = 7 mm/s; **(B)** *k*_*l*_ = 0.75 mm/(N·s), *V*_*lm*_ = 7 mm/s; **(C)** *k*_*l*_ = 0.75 mm/(N·s), *F*_*i*_ = 10 N [*F*_*i*_, unit: N; *V*_*lm*_, unit: mm/s; *k*_*l*_, unit: mm/(N·s)].

Figure 9 shows the MCF’s feature values and the participation score of Subject No. 1 in the MOTOmed mode under different FPVC strategy parameters. When *F*_*i*_ and *V*_*lm*_ are constant: *F*_*i*_ = 10 N and *V*_*lm*_ = 7 mm/s (Figure 9A), ZC values are 160, indicating that the subject has completed 80 cycles of MOTOmed training. MAV, RMS and VAR increase with the decrease of *k*_*l*_; When *k*_*l*_ = 0.5 mm/(N·s), MAV, RMS and VAR achieve maximum values, and the participation score is 5, indicating that the subjective feeling is frustration. When *k*_*l*_ and *V*_*lm*_ are constant: *k*_*l*_ = 0.75 mm/(N·s) and *V*_*lm*_ = 7 mm/s (Figure 9B), MAV, RMS, VAR increase with the increase of *F*_*i*_, and ZC value remains unchanged. When *F*_*i*_ = 18 N, the objective indicators achieve the maximum values, and the participation score is 4. When *k*_*l*_ and *F*_*i*_ are constant: *k*_*l*_ = 0.75 mm/(N·s) and *F*_*i*_ = 10 N (Figure 9C), MAV, RMS and VAR increase with the decrease of *V*_*lm*_. When *V*_*lm*_ = 3 m/s, MAV, RMS and VAR achieve maximum values, and the participation score is 4. When *V*_*lm*_ = 11 m/s, MAV, RMS and VAR achieve minimum values, and the participation score is 2.

**FIGURE 10:**
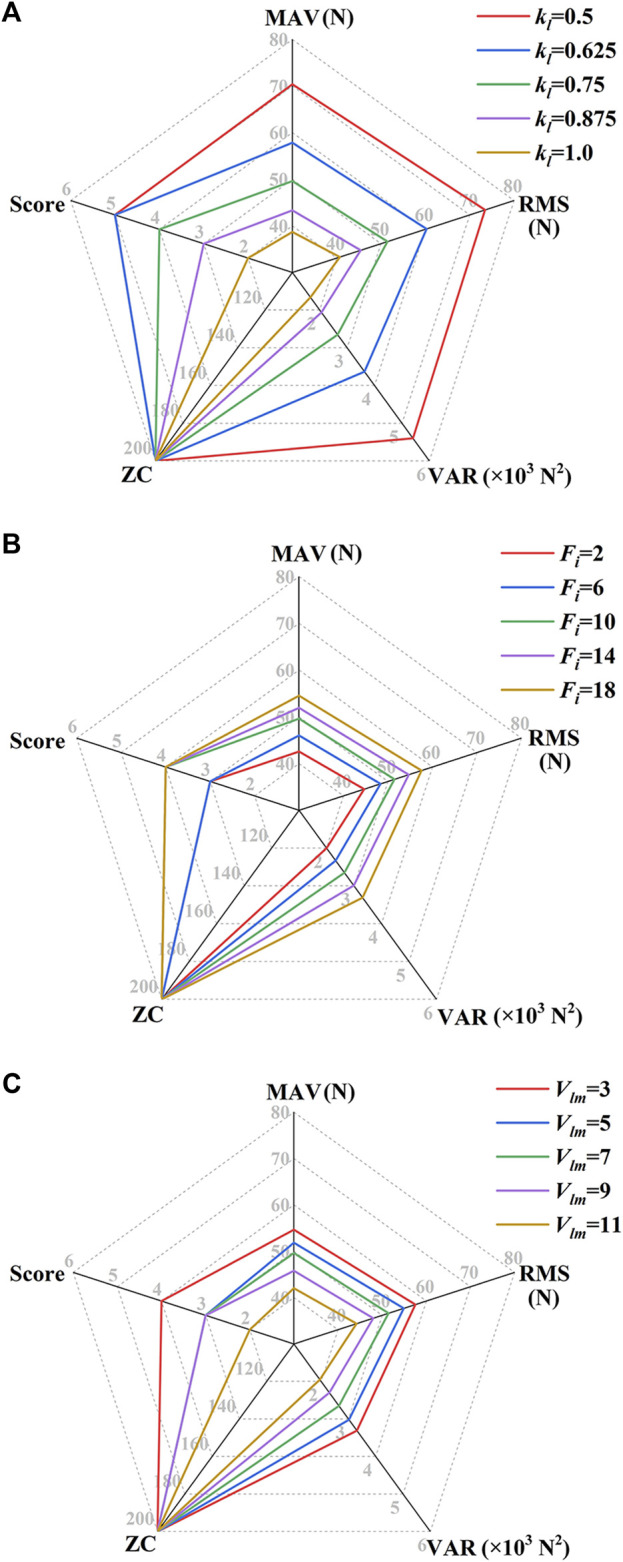
MCF’s feature values and the participation score of Subject No. 1 in the CPM mode under different FPVC strategy parameters. **(A)** *F*_*i*_ = 10 N, *V*_*lm*_ = 7 mm/s; **(B)** *k*_*l*_ = 0.75 mm/(N·s), *V*_*lm*_ = 7 mm/s; **(C)** *k*_*l*_ = 0.75 mm/(N·s), *F*_*i*_ = 10 N [*F*_*i*_, unit: N; *V*_*lm*_, unit: mm/s; *k*_*l*_, unit: mm/(N·s)].

Figure 10 shows the MCF’s feature values and the participation score of Subject No. 1 in the CPM mode under different FPVC strategy parameters. When *F*_*i*_ and *V*_*lm*_ are fixed: *F*_*i*_ = 10 N and *V*_*lm*_ = 7 mm/s (Figure 10A), the ZC value is 200, indicating that the subject has completed 100 cycles of CPM training. MAV, RMS and VAR have similar change laws as in the MOTOmed mode. When *k*_*l*_ and *V*_*lm*_ are constant: *k*_*l*_ = 0.75 mm/(N·s) and *V*_*lm*_ = 7 mm/s (Figure 10B), the ZC value is 200. When *F*_*i*_ = 18 N, MAV, RMS and VAR achieve maximum values, and the participation score is 4. When *k*_*l*_ and *F*_*i*_ are constant: *k*_*l*_ = 0.75 mm/(N·s) and *F*_*i*_ = 10 N (Figure 10C), the ZC value is also 200. When *V*_*lm*_ = 3 mm/s, the objective indicators achieve maximum values, and the participation score is 4.

Based on the above experimental results, the mapping relationship between the objective indicators (the feature values of the MCF signal) and the subjective indicators (participation scores given by the subjects) was studied to realize the prediction from the objective indicators to the subjective indicators. The particle swarm optimization-backpropagation (PSO-BP) algorithm was selected for the regression prediction of active participation. The algorithm parameter setting is shown in Table 3. Three subjects participated in the active training of the MOTOmed and CPM modes under different control strategy parameters. The feature values of MCF were taken as the training set’s input parameters ***X***_***s***_, and the questionnaire scores of subjects for different training tasks were taken as the output parameters ***Y***_***s***_ of the training set.

**TABLE 3:** PSO-BP algorithm parameter setting.

Parameter	Parameter value
Training number	1000
Learning rate	0.01
Minimum error	1 × 10^−5^
Momentum factor	0.01
Minimum gradient	1 × 10^−6^
Swarm size	30
Space dimension	82
Maximal number of iterations	100
Inertia weight	0.9
Acceleration coefficients (*c*_1_, *c*_2_)	(2, 2)

**FIGURE 11:**
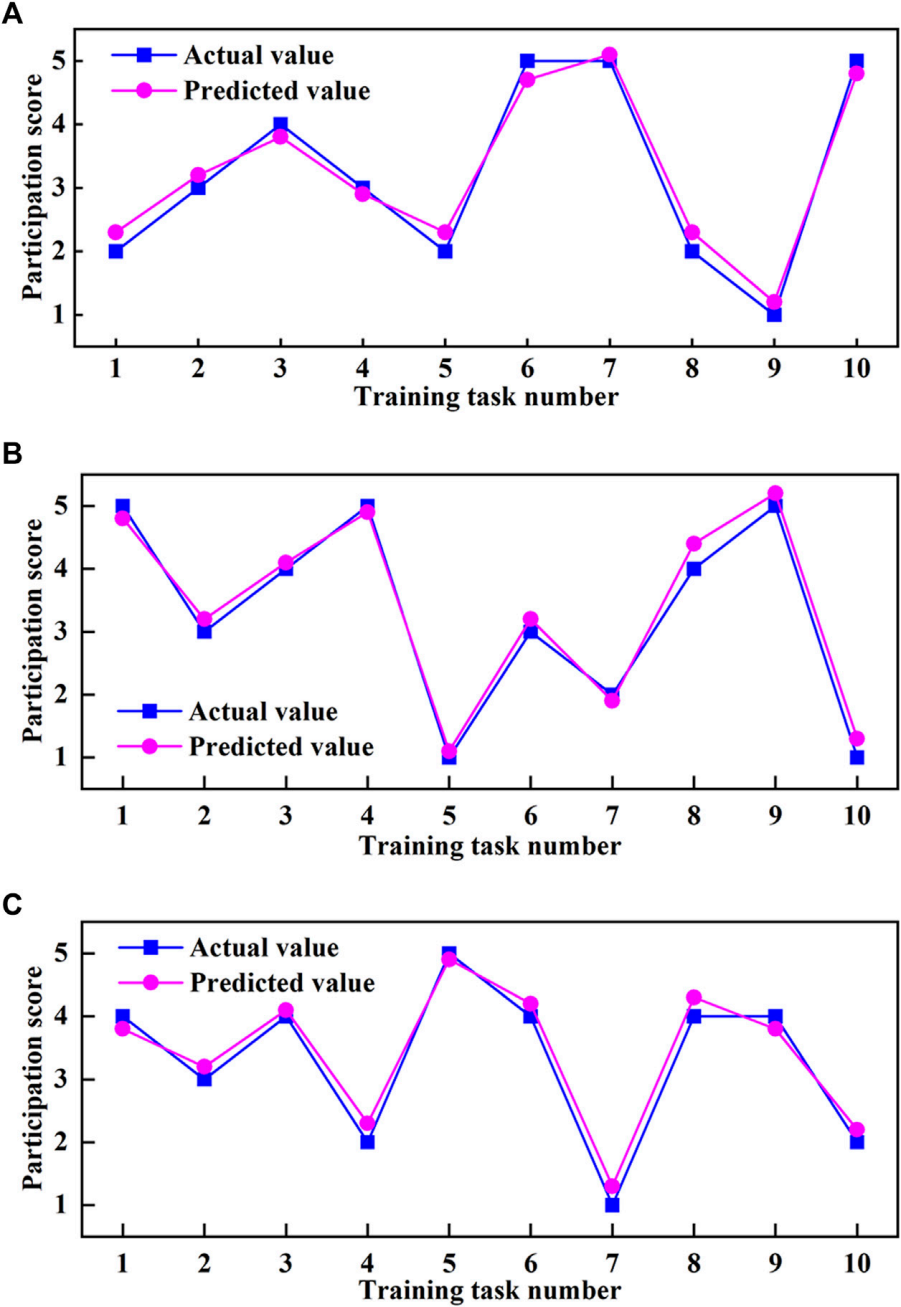
Comparison between the actual value and the predicted value of active participation score. **(A)** Subject No. 1. **(B)** Subject No. 2. **(C)** Subject No. 3.

Each subject participated in 10 groups of training under different control strategy parameters. After the experiments, they took the questionnaire survey. The feature values of MCF were taken as input parameters ***X***_***l***_ of the testing set, and the active participation scores of the questionnaire were taken as the actual output parameters ***Y***_***l***_ of the testing set. Using the trained prediction model of active participation, the predicted values ***Y***_***p***_ were predicted from the input parameters ***X***_***l***_. The comparison between the actual value and the predicted value of the testing sets for different subjects is shown in Figure 11.

The participation score of the subject in the testing set is an integer score of “1, 2, 3, 4, 5”. Because the subjects have different evaluation criteria for participation, the dynamic trends of training data are also different. The experimental results show that the active participation scores predicted by the PSO-BO algorithm are close to the actual values. If the absolute error value between the actual value and the predicted value is less than 0.25, it is regarded as accurate; Otherwise, it is regarded as inaccurate. Then the prediction accuracy rate for Subject No. 1 is 60% (Figure 11A), that for Subject No. 2 is 80% (Figure 11B), and that for Subject No. 3 is 70% (Figure 11C). If the absolute error value between the actual value and the predicted value is less than 0.5, it is regarded as accurate; Otherwise, it is regarded as inaccurate. Then the prediction accuracy for three subjects can reach 100%. The above results show that subjects’ active participation in training tasks can be predicted from the MCF’s feature values, and the prediction accuracy can meet the prediction requirements from objective feature values to subjective indicators.

## 4 Conclusion and discussion

In this paper, a force/position-based velocity control strategy was proposed for HE-LRR to meet the demands of trajectory tracking effect and active participation of lower limb rehabilitation robots. The end effector’s velocity planning was introduced in detail. Experimental studies were carried out on the control strategy with the following conclusions:

(1) The trajectory measurement experiments of HE-LRR were carried out under two training modes. The results showed that the end effector could approach the reference trajectory in a short time when the starting points of the end effector were different (inside the circular trajectory, outside the circular trajectory, above the linear trajectory, below the linear trajectory), which proved that the FPVC strategy is beneficial for subjects to achieve active rehabilitation training under accurate trajectories.
(2) The force and velocity measurement experiments of HE-LRR were carried out in two training modes. The results showed that the actual velocity of the end effector possessed good following performance compared with the desired velocity, which reflected that the robot could adapt to the changes of MCF, and proved the rationality of velocity planning in the FPVC strategy.
(3) Active participation experiments were conducted under different control strategy parameters, and the prediction of the active participation was performed using the PSO-BP algorithm. The results showed that the active participation of subjects could be adjusted by the control strategy parameters, and the active participation score could be predicted accurately from the MCF’s feature values.

Although the rationality and feasibility of the FPVC strategy have been experimentally verified on the HE-LRR system, there are still some things that could be improved in the research work. For example, the FPVC strategy was mainly validated on the end-effector lower limb rehabilitation robot under MOTOmed and CPM modes, which are training modes in the sagittal plane, and the experimental validation of the FPVC strategy was conducted by recruiting a series of healthy subjects. Our future research work will mainly focus on carrying out three-dimensional spatial trajectory verification and on the exoskeleton-type lower limb rehabilitation robot to improve the robot’s functionality and practicality, and conducting clinical experiments to study patients’ experience and active participation under the FPVC strategy.

## Data Availability

The raw data supporting the conclusion of this article will be made available by the authors, without undue reservation.
